# Testosterone Therapy in Advanced Prostate Cancer

**DOI:** 10.1089/andro.2021.0035

**Published:** 2022-12-22

**Authors:** Emily Chedrawe, Aditya Sathe, Josh White, Jesse Ory, Ranjith Ramasamy

**Affiliations:** 1 Department of Urology, Dalhousie University, Halifax, Canada; 2 Health Science Center College of Medicine, University of Tennessee, Knoxville, Tennessee, USA; 3 Department of Urology, University of Miami, Miami, Florida, USA

**Keywords:** testosterone therapy, advanced prostate cancer, ADT

## Abstract

Androgen deprivation therapy is a mainstay of advanced prostate cancer (PCa) but the resulting low testosterone levels leave men susceptible to a multitude of adverse effects. These can include vasomotor symptoms, reduced sexual desire and performance, and mood changes. Testosterone therapy (TTh) in advanced PCa has historically been contraindicated since Huggins and Hodges reported that testosterone activates PCa. Although TTh has been demonstrated to be safe in patients who have undergone treatment for localized PCa, there is extremely limited evidence on its safety in advanced PCa. Despite the lack of evidence, some men with advanced PCa still inquire about TTh, and recent publications have described its use. In this article, we review the potential implications of TTh in men with advanced PCa, defined here as biochemical recurrence after localized therapy or metastatic PCa that is either hormone sensitive or castration resistant.

## Introduction

Testosterone and its relationship with prostate cancer (PCa) progression were first implicated by Drs. Charles Huggins and Clarence Hodges in 1941.^1^ Testosterone has since frequently been suggested to fuel PCa. Researchers have since found that the relationship between androgens and PCa is more complicated, and likely not linear.^2^ For instance, men with the lowest levels of testosterone were found to be at increased risk of biopsy proven PCa compared with other hypogonadal men.^3^ Furthermore, hypogonadal men who are on testosterone do not appear to have a higher risk of *de novo* PCa.^4^ And, in men with localized PCa and curative treatment, multiple studies have shown that giving testosterone therapy (TTh) does not appear to increase the risk of disease progression or recurrence.^5–7^

Although the U.S. Food and Drug Administration maintains the warning that androgens may increase the risk of PCa and lists known or suspected carcinoma of the prostate as a contraindication, the American Urological Association (AUA) provides a strong recommendation that patients should be informed that there is a lack of evidence to support the previously believed theory that TTh is linked to the development of PCa.^8^ A Canadian Survey of Urologists found a majority of physicians consider TTh safe in men who underwent radical prostatectomy, radiation therapy, brachytherapy at 96%, 84%, and 86%, and 65% believe it is safe for men on active surveillance.^9^

Although attitudes regarding the safety of TTh and localized PCa have changed recently, this survey suggests that urologists believe TTh may be less safe in men with PCa with a prostate *in situ*. The use of TTh in advanced PCa, defined as biochemical recurrence after localized therapy (BCR) or metastatic PCa that is either hormone sensitive (mHSPC) or castration resistant (mCRPC), is a much more controversial topic and not recommended by current guidelines.^9^

Nearly half of all men with advanced PCa report high levels of distress and poor quality of life (QoL).^10^ Low sexuality and hypogonadal symptoms, a consequence of primary treatments and androgen deprivation therapy (ADT), are consistent drivers of poor life satisfaction.^11,12^ When compared with individuals with localized PCa, those with advanced disease on ADT consistently demonstrate greater severity of sexual dysfunction, hot flashes, low energy, and weight gain.^11^ Furthermore, men with advanced disease or on ADT are less likely to be offered interventions for sexual dysfunction.^11^ However, it is unclear whether or not using testosterone in advanced PCa functions the same way as using TTh for men with localized PCa.

Scarce literature is present regarding TTh and advanced PCa. Few researchers have explored this topic since Fowler and Whitmore’s study from 1981 showing 45 out of 67 men with metastatic PCa had unfavorable responses to exogenous testosterone and that the response occurred rapidly within 30 days.^13^ The safety of TTh in this population is unknown, and by current standards is considered unsafe. Despite this, a research group from Boston recently published a study investigating the safety of TTh in advanced PCa in a cohort of 22 men.^12^ Our objective is to review the use of TTh in advanced PCa, its implications, and the potential issues of hypogonadism in this disease state.

### Impact of Low Testosterone and Hypogonadal Symptoms in Patients on ADT

As men age they exhibit decreasing levels of total and free testosterone at a rate of 0.8–2% per year.^14^ The combination of low total testosterone (<300 ng/dL) and clinical symptoms in aging men creates a syndrome named late onset hypogonadism. Hypogonadism is associated with both decreased QoL and metabolic consequences. Symptoms include reduced sexual desire, erectile dysfunction, fatigue, depressed mood, and hot flashes.^15^ Metabolically, low testosterone has been linked to obesity, cardiovascular events,^16^ type 2 diabetes mellitus,^17^ and decreased bone mineral density.^18^

The goals of TTh are to restore serum testosterone levels to within the midnormal physiological range, generally considered to be between 400 and 700 ng/dL, and to improve symptoms in hypogonadal men.^15^ TTh has been shown to have the greatest improvement in increasing sexual thoughts and increased frequency, duration of erections, depressive symptoms, and bone mineral density.^8^ Despite these reported improvements, research has not demonstrated a clinically significant improvement on QoL.^8^

Men treated for PCa who receive ADT experience the same constellation of issues as men with hypogonadism. The most common form of ADT is luteinizing hormone-releasing hormone (LHRH) agonists and antagonists, which achieve castrate levels (<50 ng/dL) of circulating testosterone through modulation of the hypothalamus–pituitary–gonadal axis. Even temporary ADT has a profound ability to suppress testicular production of testosterone and the effect can be long lasting.

Nascimento et al. reported a study of 307 men who received ADT for primary PCa, 25% of whom remained below normal testosterone levels (>300 ng/dL) and 10% of whom remained below castrate levels (50 ng/dL) as far as 2 years after cessation of therapy.^19^ A significant challenge in using ADT as the first-line treatment for advanced PCa is finding the balance between improving survival while mitigating the burden of hypogonadal symptoms.

ADT has pros and cons. Men with advanced PCa treated with early ADT demonstrated a significant increase in 5-year (88% in ADT group vs. 78% in control group, *p* = 0.04) and 10-year survival (53% in ADT group vs. 38% in control group, *p* < 0.004).^20,21^ Androgen deprivation is also associated with decreased rates of morbidity such as cord compression, ureteral obstruction, and extraskeletal progression.^22^ Although ADT can be used as monotherapy for advanced PCa, the AUA guidelines have recommended the use of ADT with an androgen receptor (AR)-axis-targeted therapy based on evidence from multiple clinical trials demonstrating improved survival using combination therapy for mHSPC, nmCRPC, and mCRPC.^23,24^

Unfortunately the magnitude of ADT side effects can be significant. Short-term adverse effects of ADT, namely the vasomotor symptoms, are thought to arise from the increased release of hypothalamic catecholamines in response to decreased LH and FSH levels.^25^ The increase in catecholamines can cause a multitude of symptoms including sweats, hot flashes, headaches, and palpitations. Sexual dysfunction, such as loss of libido, mood swings, fatigue, and erectile dysfunction, affect 90% of patients on ADT.^26^ Challapalli et al. described the incidence of hypogonadal symptoms in a cohort of 250 patients actively receiving ADT for primary PCa.

A scoring system from Grades 1 to 4 was utilized to quantify severity of symptoms with Grade 4 defined as bothersome toxicity with the need for medical intervention. Overall, 38.4% of patients had Grade 3 and Grade 4 symptoms with 20% of the Grade 4 cohort requiring cessation of ADT.^27^ Of these symptoms, the most common were hot flashes and sweats followed by fatigue and changes in sleep quality. Independent risk factors for the severity of hot flashes and sweats included younger age and Afro-Caribbean race.

With respect to metabolic consequences, a large U.S. study showed patients on ADT were 44% more likely to develop diabetes with a 16% increased risk of sudden cardiac death compared with patients with PCa not receiving ADT.^28^ Overall, literature reports a 14–39% increased risk of fracture in patients exposed to ADT compared with unexposed controls.^29,30^

Alternatives to TTh for treatment of hypogonadal sequelae of ADT have been explored such as manipulating ADT administration protocols, using therapies to target specific side effects, and nonpharmacology therapies. Traditionally ADT is administered continuously, however, intermittent administration is supported for men who have BCR or early mHSPC.^23,31^ Intermittent ADT involves cyclic administration of ADT to restore QoL measures and minimize long-term morbidity of ADT, without sacrificing survival outcomes.^32^ Benefits with respect to QoL, sexual, mental, and physical measures in intermittent ADT are variable among numerous studies.^31^

Hussain et al. demonstrated that intermittent ADT is associated with significantly less erectile dysfunction, better mental health outcomes, and small improvements in QoL compared with continuous therapy in men with metastatic PCa in a randomized control trial involving 3040 patients with metastatic HSPC.^33^ These benefits were not consistent across all follow-up time points and seemed to align with breaks in ADT when testosterone levels had recovered. Multiple clinical trials support the safety of intermittent ADT finding that oncological outcomes, such as PSA progression and overall survival, are equivalent compared with continuous therapy in locally advanced and mHSPC.^31^

Relugolix, an oral gonadotropin-releasing hormone antagonist, has been studied as another alternative for achieving androgen deprivation instead of the commonly used LHRH agonists/antagonists. The HERO study, a phase 3 clinical trial comparing 622 patients receiving relugolix compared with 308 patients receiving leuprolide, demonstrated significantly shorter time to reach castration levels and a greater ability to maintain castration level of testosterone.^34^

Furthermore, discontinuation of this short-acting ADT demonstrated a greater potential of recovering testosterone levels to normal range, as 54% had testosterone recovery after 90 days of discontinuation, compared with only 3% in the leuprolide group. Although ADT is not typically stopped when treating advanced PCa, relugolix may be a better alternative to traditional ADT in the event men do not tolerate the symptoms of medical castration and desire recovery to normal levels.

Other nonhormonal strategies may be used to mitigate the side effects of ADT. These include treatments such as dietary supplements, bisphosphonates, and antidepressants. Research also demonstrates improvements in physiological decline through diet and exercise interventions.^35^ Nonpharmacological therapies, such as patient education programs, may improve the psychological sequelae of ADT.^36^

### Biochemical Differences of Advanced PCa That Alter Androgen Response

The effect of TTh on benign prostate tissue was studied in a randomized control trial involving 44 men with symptomatic hypogonadism.^37^ They found that administration of exogenous testosterone to achieve physiological levels had little impact on prostatic tissue with respect to prostatic androgen levels, markers of cell proliferation, or angiogenesis. They concluded that the prostate is able to sequester sufficient levels of androgens despite low serum levels. To explain, a “saturation model” was suggested by Morgentaler and confirmed by Rastrelli et al.,^38^ proposing that after a certain low level (~240–250 ng/dL in humans), prostatic ARs will become saturated, and no longer respond to additional testosterone.^2^ This theory has further been applied to explain the safe use of TTh in localized PCa.

However, it is important to consider the biochemical difference of advanced PCa to nonadvanced PCa when evaluating the safety of TTh. Mutations in the AR and complementary pathways are not typically seen in primary cancer cells but found in 70% of CRPC,^39^ and allow the AR to drive tumorigenesis despite castrate testosterone levels. The most common mutation is amplification of the AR, leading to AR overexpression, increased androgen binding potential, and less receptor specificity to steroid ligands.^40,41^

The result is a more heterogeneous population of cells within the same tumor. Some populations may be more sensitive to circulating androgens, whereas others proliferate independently. AR-axis-targeted therapies were developed to directly target the AR and DNA repair pathways, however, these newer drugs do not mitigate the need for ADT. Research involving monotherapy with ARAT has been shown to be less effective than androgen deprivation in metastatic PCa, demonstrating the necessity of low serum testosterone in advanced cancer treatment.^42^

In addition, studies show targeting serum testosterone <20 ng/dL for ADT, instead of the traditional target of 50 ng/dL, leads to significantly better biochemical relapse-free survival,^43^ significant lower risk of death,^44^ and better cancer-specific survival.^45^ Combining these two modalities allows for complete androgen blockade and improved oncological outcomes.^46^ These unique properties of advanced PCa render the assumptions of the saturation hypothesis oversimplified and likely invalid.

In the clinical setting, our understanding of how testosterone and advanced PCa interact has been addressed by a few clinical trials with the introduction of bipolar androgen therapy (BAT) in 2015.^47^ BAT is a therapeutic approach developed to prolong hormone sensitivity and prevent mutational pressures in metastatic PCa through oscillation between castration levels of testosterone using ADT and periods of supraphysiological levels of testosterone using testosterone injections.^48^ The protocol involves 400 mg injections of testosterone cypionate every 28 days while patients remain on continuous ADT.^48^

In a pilot study, 16 men with CRPC were subjected to this BAT protocol in addition to oral etoposide on days 1–14. Findings demonstrated 50% had radiological response and a third of the patients had a 50% reduction in PSA and improvements in QoL. Furthermore, three men regained response to previously failed antiandrogen therapy.^48^ This protocol was also studied in mHSPC in the BATMAN study, which included 29 patients, with similar outcomes.^49^ The proposed mechanism suggests the mutated pathways activated by androgen deprivation are turned off when testosterone levels are high. In addition, high levels of androgens cause DNA strand breakage in rapidly dividing cells.^50^

The most recent research in this field has integrated and compared BAT with standard of care therapies. One study showed promising preliminary findings using combination BAT with olaparib, a poly ADP-ribose polymerase inhibitor, to target different pathological pathways in patients with mCRPC. They found PSA decreased >50% in 14 out of 30 participants.^51^ In the TRANSFORMER study by Denmeade et al., BAT was compared with the antiandrogen enzalutamide in a clinical trial on 195 patients with mCRPC progressing on abiraterone.

Although they were unable to demonstrate superiority of BAT over enzalutamide, they did show no significant difference in clinical or radiographic progression-free survival with BAT. Furthermore, BAT was associated with lower rates of fatigue and sexual dysfunction, and more favorable QoL outcomes.^52^ Although BAT has not been widely taken up into practice, it may provide similar survival outcomes with improved hypogonadal symptoms in men who are not interested in conventional treatment protocols.

Another challenge within this area of research is increased heterogeneity of what constitutes advanced PCa. Progression from BCR to CRPC occurs quickly as these tumors are quick to grow and mutate. The mean time for HSPC to develop to castrate resistance is 12–48 months.^53^ This demonstrates the aggressive ability for these cells to undergo mutation. The increased complexity of advanced disease makes evaluating the safety of TTh in this population more difficult.

### What Are the Known Clinical Risks and Benefits of TTh in Patients with Advanced PCa?

Currently insufficient data are available to make conclusions regarding the safety and therapeutic benefit of TTh in advanced PCa. However, many barriers exist that make conducting high-powered randomized controlled trials with appropriate follow-up impractical. The only clinical study to date from Morgentaler et al. lays some groundwork toward answering these questions, however, careful interpretation of the results must be taken given the many study limitations. His research group presented a retrospective nonrandomized observational study of 22 men with either BCR, metastatic PCa, or adjuvant ADT after local treatment who strongly sought TTh for symptom relief even after being counselled of the risks of progression or death.^12^

Inclusion criteria included hypogonadal symptoms and serum testosterone <350 ng/dL. TTh formulations including testosterone injections, gels, subcutaneous pellets, and the modified BAT protocol varied in the cohort. Subjective symptom response was recorded from structured interview questions during follow-up. Disease outcomes were monitored at variable intervals among the cohort and included PSA and imaging studies. Although the authors conclude all participants had “considerable subjective improvement in quality of life without rapid progression, morbidity or death,” there are considerable design flaws and potentially overlooked results.

Overall, the study had a lack of consistency. First, there was a large degree of heterogeneity in the clinical staging and tumor burden across the study groups. Multiple forms of TTh were utilized and on average participants had a treated testosterone of 1011–1062 ng/dL, which is much higher than the typical treatment range of 400–700 ng/dL.^15^ Response to treatment was measured qualitatively, without a validated tool allowing for significant bias. The authors did not measure pretreatment or post-treatment symptom burden, making it difficult to appreciate the true benefit given the potentially large risk these men are taking. Furthermore, follow-up imaging was only available for 10 out of 14 men with metastatic PCa within a year of starting therapy, of whom 3 showed progression.

In addition, 7 of the 14 men were not on ADT at the time of consultation, indicating that this study is largely evaluating men not on standard of care for advanced PCa. Lastly, the lack of matched controls makes it difficult to appreciate the significance of the results as progression of disease to some extent is expected in advanced PCa. Comparing this population’s outcomes with the natural course of advanced PCa is challenging, given both the heterogeneity in this study and in the disease itself.

Even in this heterogeneous group, safety was a concern. Overall, 10 participants discontinued TTh, 7 of whom reported worsening bone scan, bony pain, rising PSA, or positive node biopsy. Eight participants continued TTh, despite four having reported worsening of disease on imaging. The study reported three deaths in the follow-up period, not including a participant with BCR who died of metastatic PCa 6 years after discontinuing TTh due to a large rise in PSA.

Of note, the median PSA doubling time (PSADT) was 8.9 and 4.4 months in the BCR and metastatic groups, respectively. Clinical guidelines report that PSADT of <12 months in men with BCR indicates higher risk of developing metastasis.^23^ Furthermore, PSA rose >10 ng/mL in patients with a mean testosterone level >200 ng/dL, a finding that does not align well with the currently proposed “saturation model” for localized PCa.

Despite these issues, the findings are an important preliminary exploration in the development of a more complete biochemical model to understand advanced PCa. This study does highlight that there is a subset of patients who, despite being well informed of the serious risks of TTh, are willing to make such trade-offs for possible improvements in QoL. For some of the participants, the extent of improvement in vigor was significant enough that they were able to forego using a walker. Even though safety must be of paramount concern, it would be a disservice to patients to ignore the needs of this subset of patients. Future studies should attempt to investigate the burden of hypogonadal symptoms on QoL relative to goals of care in men with advanced PCa.

## Conclusion

Given the current understanding of advanced PCa response to androgens, the importance of ADT in PCa treatment, and morbidity and mortality associated with using TTh in advanced PCa, the potential risks of adding TTh in this disease state are significant. Despite this, the impact of hypogonadism on QoL in this cohort is significant, and therapies with fewer side effects or safe treatment option are needed. As newer drugs for advanced PCa develop, we must be cautious not to minimize the importance of QoL in the pursuit of improved survival outcomes. Prospective observational studies in each subtype of advanced PCa would be needed to accurately outline the safety and risks of TTh in this cohort.

Nonetheless, a subset of patients may have alternative goals of care and are willing to take these risks for possible improvements in vigor, sexuality, and mood. Alternatively, we have outlined a number of potentially safer strategies, such as intermittent ADT, BAT, and short-acting ADT with relugolix, which could improve hypogonadal symptoms without compromising morbidity and mortality of advanced PCa. Ultimately, considering our current understanding, TTh has the potential to significantly worsen prognosis for patients with advanced PCa and is contraindicated for this population. Physicians should continue to provide education for patients that is sufficient for them to make their own decisions.

## Abbreviations Used

ADT: androgen deprivation therapy
AR: androgen receptor
AUA: American Urological Association
BCR: biochemical recurrence after localized therapy
LHRH: luteinizing hormone-releasing hormone
mCRPC: metastatic castration-resistant prostate cancer
mHSPC: metastatic hormone-sensitive prostate cancer
PCa: prostate cancer
PSADT: PSA doubling time
QoL: quality of life
TTh: testosterone therapy
